# A Germany-wide survey of caregiving professionals on climate change and mental health of children and adolescents - factors influencing their relevance rating of extreme weather event associated mental health impairments

**DOI:** 10.1186/s12889-023-17576-6

**Published:** 2024-01-08

**Authors:** Annika Hieronimi, Fiona O’Reilly, Michael Schneider, Inga Wermuth, Gerd Schulte-Körne, Lena Lagally, Stephan Bose-O’Reilly, Erik Danay

**Affiliations:** 1 Institute and Clinic for Occupational, Social and Environmental Medicine, University Hospital, LMU Munich, Munich, Germany; 2 Institute of Sociology, Ludwig-Maximilians-University Munich, Munich, Germany; 3 Department of Child and Adolescent Psychiatry, Psychosomatics and Psychotherapy, University Hospital, LMU Munich, Munich, Germany; 4 Institute of Public Health, Medical Decision Making and Health Technology Assessment, Department of Public Health, Health Services Research and Health Technology Assessment, Medical Informatics and Technology, UMIT University of Health Sciences, Hall in Tirol, Austria

**Keywords:** Extreme weather events, Mental health, Children and adolescents, Medical and therapeutical staff, School and pedagogical staff, Relevance assessment, Influencing factors

## Abstract

**Introduction:**

Climate change presents a significant risk for the mental and physical health of young people. In order to identify and properly care for potential mental health impairments from extreme weather events, the relevance of these impairments must be assessed as high by the professional groups providing care for children and adolescents. This raises the question of which factors influence the individual relevance assessment of caretaking professionals?

**Methods:**

Data was collected creating and conducting a Germany-wide online questionnaire via LimeSurvey. The questionnaire was addressed to professionals providing care for children and adolescents, in this case medical and therapeutic personnel as well as school and pedagogical personnel. Professional associations, chief physicians and school principals were contacted as multipliers and asked to forward the questionnaire to their members and staff. The data was analyzed using the R statistical software, and multiple linear regressions were performed to test the hypotheses.

**Results:**

Overall, 648 questionnaires were taken into analysis. Approximately 70% of the participants considered climate change-induced impacts on the mental health of children and adolescents due to extreme weather events as relevant. Experiencing heat, storm, heavy precipitation, flood/flooding, and/or avalanches/mudflows made a modest yet significant contribution to explaining higher relevance assessments. In contrast, there was no evidence to suggest that an urban working environment increases the relevance assessment.

**Conclusion:**

The described influence of experiencing extreme weather events should not be regarded as the sole factor leading to higher relevance ratings. A more comprehensive understanding of the factors influencing relevance assessments is necessary to address key aspects of risk communication and increase risk awareness.

**Supplementary Information:**

The online version contains supplementary material available at 10.1186/s12889-023-17576-6.

## Introduction

The effects of climate change represent one of the greatest threats to humanity [1]. Particular attention should be paid to the impacts of extreme weather events (EWE) on both physical and mental health. In contrast to the effects of EWE on physical health, such as injury and death, the effects on mental health have been little researched [2]. In this context, children and adolescents are a particularly vulnerable group that will be affected by EWE much more often in the future [3].

### What are the known impacts of EWE on the mental health of children and adolescents?

Known mental health impacts from climate change and especially EWE are diverse, ranging from emotions as an adequate response to abnormal situations to diagnosable illnesses [4–7].

Short-lasting EWE, such as storms or heavy precipitation, can impact mental health. After experiencing an EWE post-traumatic stress symptoms (PTSS) or post-traumatic stress disorders (PTSD) are seen in children and adolescents [8]. In Germany approximately 5–6% of PTSS and PTSD in children and adolescents can be attributed to experiencing an EWE [9]. International studies indicate a prevalence of PTSD following an EWE of 5–43% [10]. Risk factors for the development of PTSD include physical injury from the EWE, death of loved ones, and pre-existing mental illness in children and adolescents or their caregivers [7, 11]. One of the reasons for the high vulnerability of children and adolescents is that they have less life experience, resulting in a lower ability to process the events that have happened [12]. In contrast there are protective factors including the opportunity to relocate with their parents and the involvement of children and adolescents in cleanup activities after EWE [7, 13]. Additionally returning into the school system as soon as possible after the event improves the children’s mental wellbeing [14].

For long-lasting EWE, such as extreme heat, there is limited literature on its effects on child and adolescent mental health. In a U.S. study, the increased number of emergency department visits due to mental health impairment among children and adolescents was detected during higher temperatures [15]. Other studies have shown an increase aggressive behavior and elevated suicide rates associated with heat in children and adolescents [16, 17].

Perceptions of climate change are highly subjective and depend on sociocultural background as well as prior individual experiences [1]. Children and adolescents are becoming increasingly concerned with the issue of climate change, triggering feelings of anxiety and helplessness in some [4]. In a study from the United Kingdom, 74% of young people said they were concerned about the impacts of climate change [18].

### Why should caretaking professionals have a responsibility to know about the mental health implications of climate change for children and adolescents and to act on that knowledge?

In the professional regulation for physicians working in Germany it is written that it is their responsibility “to participate in the preservation of the natural basis of life for the health of people” [19]. In addition to the general knowledge of all physicians about the effects of climate change, pediatricians in particular should learn to provide proactive advice to prevent climate change-related health threats [20]. However, referring patients with mental health problems to the appropriate professionals is the responsibility of all practicing physicians [20].

Teachers, on the other hand, do not have a specific professional code that they have to follow, but there are various guiding principles for pedagogical professions. These include, for example, the Socratic Oath by von Hentig, which states that teachers must “advocate for the physical and mental integrity of the child” [21]. In Zierer’s contemporary interpretation, this promise is still a central part of the oath [22]. The duty of supervision of teachers also includes the task to protect students from harm [23]. Despite these principles, teachers are not responsible for the medical care of children and adolescents due to their lack of professional training, but they can make a difference by helping in prevention [24].

For an adequate care a perceived high relevance of a topic in the group of caregiving professionals is necessary. In this case, relevance describes recognizing the consequences of climate change such as EWE as a risk to the mental health of children and adolescents. This is an important prerequisite for behavior change, implementation of adaptation measures, and planning intervention strategies [10, 25, 26].

### What factors might influence the relevance assessment of the impact of EWE on child and adolescent mental health?

There are many factors that might influence the caregiving professional’s assessment of the relevance of mental health impairments associated with extreme weather events. Two possible influencing factors are discussed below.

#### Affectedness of the personal environment by EWE

One factor that influences relevance ratings is being affected by the impacts of climate change, specifically EWE [27]. Studies in Zimbabwe, Australia, and France showed that people who have experienced EWE themselves have higher risk perceptions of climate change and show greater willingness to engage in climate change-adaptive behaviors [26, 28]. In addition, a European study showed that residential vulnerability to EWE also leads to higher risk perception of climate change [29].

In 2018, Hayes draws on Gidden’s Paradox to explain the relationship between climate change and its perception. It states that people who are not affected by immediate climate change impacts do not perceive climate change as a direct threat and do not engage in climate-sensitive behavior. However, once the impacts become noticeable and visible and people would come into action, it is too late to do anything about climate change [30].

#### Personal environment

Another influencing factor is the living and working environment. A difference between rural and urban populations is evident in terms of knowledge about climate change and its impacts. In urban areas, people tend to be more informed and obtain their knowledge about climate change from more reliable sources [31, 32]. Also, more skepticism about the relevance and in some cases even the existence of climate change was found in rural areas in Australia [33].

In contrast, higher nature awareness was found among rural populations, which is causal for the implementation of environmentally friendly behaviors [34]. In this context, increased environmental awareness is also a predictor of perceived greater risk from climate change [35].

With this in mind, the following hypotheses emerge:

The assessment of relevance of extreme weather event associated mental health impairments in children and adolescents by caregiving professionals differs depending on the following variables:

- Respondents perceive the relevance of mental health impairments of children and adolescents associated with EWE as higher if their environment is more affected by EWE.
- Respondents who work in an urban environment rate the relevance of the mental health impairments of children and adolescents associated with EWE as greater than those who work in a rural environment.

## Methods

### Creation of the questionnaire

The questionnaire was developed for this project with the help of an interdisciplinary team of scientists from pediatrics, psychiatry, pedagogy, health sciences and sociology. Questions were created after prior literature research and adapted from existing questionnaires. The questions were modified in joint discussion rounds and the questionnaire was compiled for the respective occupational groups based on their professional background.

Depending on their occupational group, participants were presented with either 32 (medical and therapeutic professionals - MTP) or 23 (school and pedagogical professionals - SPP) questions. General questions about the impact of climate change and EWE in particular on mental health and sociodemographic questions were answered by both occupational groups. MTP provided additional information about risk factors, care, and prevention. SPP, on the other hand, answered questions about prevention and the handling of mental impairments among their students.

Individual questions could be skipped by the participants. The use of different question styles was intended to increase the motivation to answer. For closed questions, participants could choose either one or more answer options (15/13 questions) or indicate their agreement with certain statements using a five-point Likert scale ranging from “not at all relevant” to “very relevant,” “does not apply at all” to “fully applies,” or “not affected” to “strongly affected” (10/5 questions). For open questions they were asked to give a statement on different topics or to elaborate on a closed question (7/5 questions).

The questions were transferred to the online application “LimeSurvey” after development. A maximum of ten minutes was estimated for completing the questionnaire. The data collection was anonymous. The participants were informed that it was possible for them to end the survey at any time. A privacy statement had to be accepted before starting the survey.

### Pretests

Before starting the online survey, seven pretests were conducted on volunteer representatives of the two occupational groups. The participants first answered the questionnaire independently to check the processing time. Then they were asked to review the questionnaire question by question using Think Aloud and Probing [36]. After each of the seven pretests, a discussion session was held to adjust questions as well as response options, to add or discard individual questions and to change the order of questions.

### Dissemination strategy

For the dissemination of the questionnaire, professional associations, as well as chief physicians of German children’s hospitals and child and adolescent psychiatries and principals of different types of public schools were contacted. They were selected as multipliers and were asked to forward the information to their members and employees. All contacted individuals received an e-mail with a brief summary of the research project, the link to complete the questionnaire and a contact option in case of further questions.

Using a publicly available list of all German pediatric clinics from the German Society for Pediatric and Adolescent Medicine (as of May 1st, 2021), an online search was conducted to generate a list of e-mail addresses of each chief physician and the corresponding secretaries. Additionally, professional associations and societies were contacted to disseminate the questionnaire.

To reach public schools in both urban and rural settings, one county and one city were randomly selected per state. The email addresses of the principals and associated secretariats of all schools in the selected locations were generated via online search and inserted into a list. Again, professional associations and unions were contacted for further dissemination of the questionnaire.

### Data collection

Data collection took place between July 27th and October 14th, 2021. After interested persons started the survey by clicking on the Lime Survey link in the received e-mail, they were guided online through the different questions. The completed questionnaires were reviewed daily during the survey period to resolve any potential problems respondents may have had in completing the questionnaire.

### Data analysis

After exporting the data from Lime Survey and data cleaning, the data was analyzed using the statistical program *R* 4.2.1 [37].

First, a descriptive analysis of the variables “relevance assessment of EWE associated mental health impairments of children and adolescents in Germany at present”, “the affectedness of the personal environment by six different EWE (heat, drought, heavy precipitation, storm, flood/flooding and avalanches/mudflows)” and the “population density of the work environment” was conducted. These variables were collected using the questions in Table 1, which were answered by both occupational groups.

**Table 1:** Questions for determining the variables

Question	Answer options
A2. In your opinion, how relevant are the following effects of climate change for child and adolescent health in Germany at the current time: c) Mental health impairment due to EWE	• Not at all relevant • Hardly relevant • Neither • Somewhat relevant • Very relevant
D4. In what environment do you work?	• Rural environment (< 5,000 inhabitants) • Small town (5,000–20,000 inhabitants) • Medium town (20,000-100,000 inhabitants) • Small metropolitan area (100,000-500,000 inhabitants) • Large metropolitan area (> 500,000 inhabitants)
D6. How affected by extreme weather events is your environment? a) Heat b) Drought c) Heavy precipitation d) Storm e) Flood and flooding f) Avalanches and mudflows	• Not affected • Rather not affected • Neither • Rather affected • Strongly affected

For hypothesis a), the influence of the affectedness of the personal environment by EWE on the relevance assessment was tested by means of a multiple linear regression.

In a first step, gender and age were included as control variables. The individuals with the gender “diverse” were excluded from this part of the statistical analysis because only two individuals belonged to this group, making the group too small for a meaningful statistical comparison.

Subsequently, to test hypothesis a), a multiple linear regression (MLR) was calculated for each of the six EWE (heat, drought, flood/flood, avalanche/mudflow, heavy precipitation, storm). The MLRs were used to test whether the predictor, in this case EWE affectedness, could predict the dependent variable of the relevance assessment. Here, the values of the standardized (std.) β indicate that if the rating of being affected by the respective EWE shifts upward by one standard deviation (SD), the relevance assessment changes by the value of the std. β. Furthermore, the MLRs tested the share that being affected by EWE has in explaining the variance of the relevance assessment beyond the variables of age and gender. This variance growth can be seen in the ∆R² data. The R² indicates the contribution of the EWE to the explanation of the variance of the relevance assessment for the control variables and the affectedness by EWE. No linearity check was performed because the predictors (affectedness by EWE) were ordinal and entered as factors.

In a further step, the chi-square difference test was used to test whether the increase in explained variance by the addition of affectedness by EWE as predictor was significant. The alpha level was set a priori at 0.05. Effect sizes were interpreted according to the cut-offs suggested by Cohen [38].

For hypothesis b), we tested whether the population density of the respondents’ work environment had an influence on the relevance assessment. For this purpose, a MLR was also calculated. The same control variables were used as for hypothesis a) and the gender “diverse” was also excluded from the evaluation. The variable “rural environment (< 5,000 inhabitants)” was set as the baseline. Everything else of the procedure was kept the same to the statistical evaluation of hypothesis a).

## Results

### Descriptive statistics

#### Sample description

A total of 648 questionnaires were included in the analysis. Previously, 272 questionnaires had to be excluded from the analysis because the filter question about the occupational group affiliation remained unanswered. Of the 648 questionnaires analyzed, 384 were for MTP and 264 were for SPP. Among the 384 survey participants from the medical and therapeutic field, the two proportionally largest groups were specialists in child and adolescent medicine (n = 129; 33.6%) and child and adolescent psychotherapists (n = 107; 27.9%). Among the SPP, teachers (n = 205; 77.7%) represented the largest proportion of participants. Further descriptive analysis of socio-demographic data can be found in the additional file 2.

#### Relevance assessment of EWE associated mental health impairments of children and adolescents in Germany at present

**Fig. 1:**
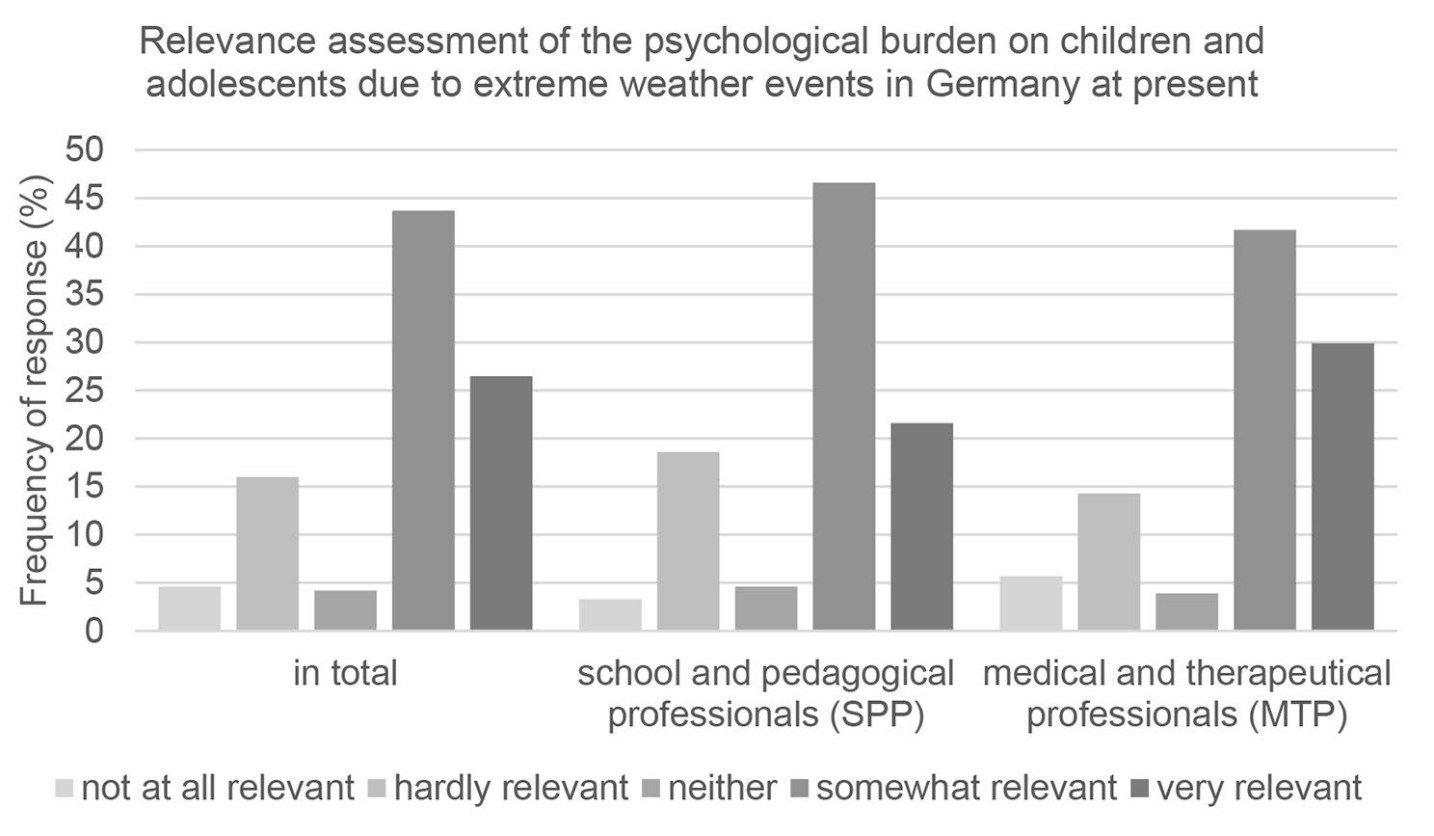
Relevance assessment of EWE associated mental health impairments of children and adolescents in Germany at present; response behavior subdivided into the occupational groups

Regardless of their occupational group, the majority of respondents assessed the relevance of mental health impairment due to EWE as somewhat or very relevant at the present time in Germany (see Fig. 1). However almost 10% more MTP opted for “very relevant” than the SPP. Among the latter, the “somewhat relevant” group was about 25% larger than the “very relevant” group. This difference was smaller among MTP (about 12%).

About 20% of the respondents, on the other hand, rated the relevance of mental health impairment caused by EWE as hardly relevant or not relevant at all. Thereby, “not at all relevant” was selected significantly less by both occupational groups than “hardly relevant”.

#### Affectedness of the personal environment by six different EWE

**Fig. 2:**
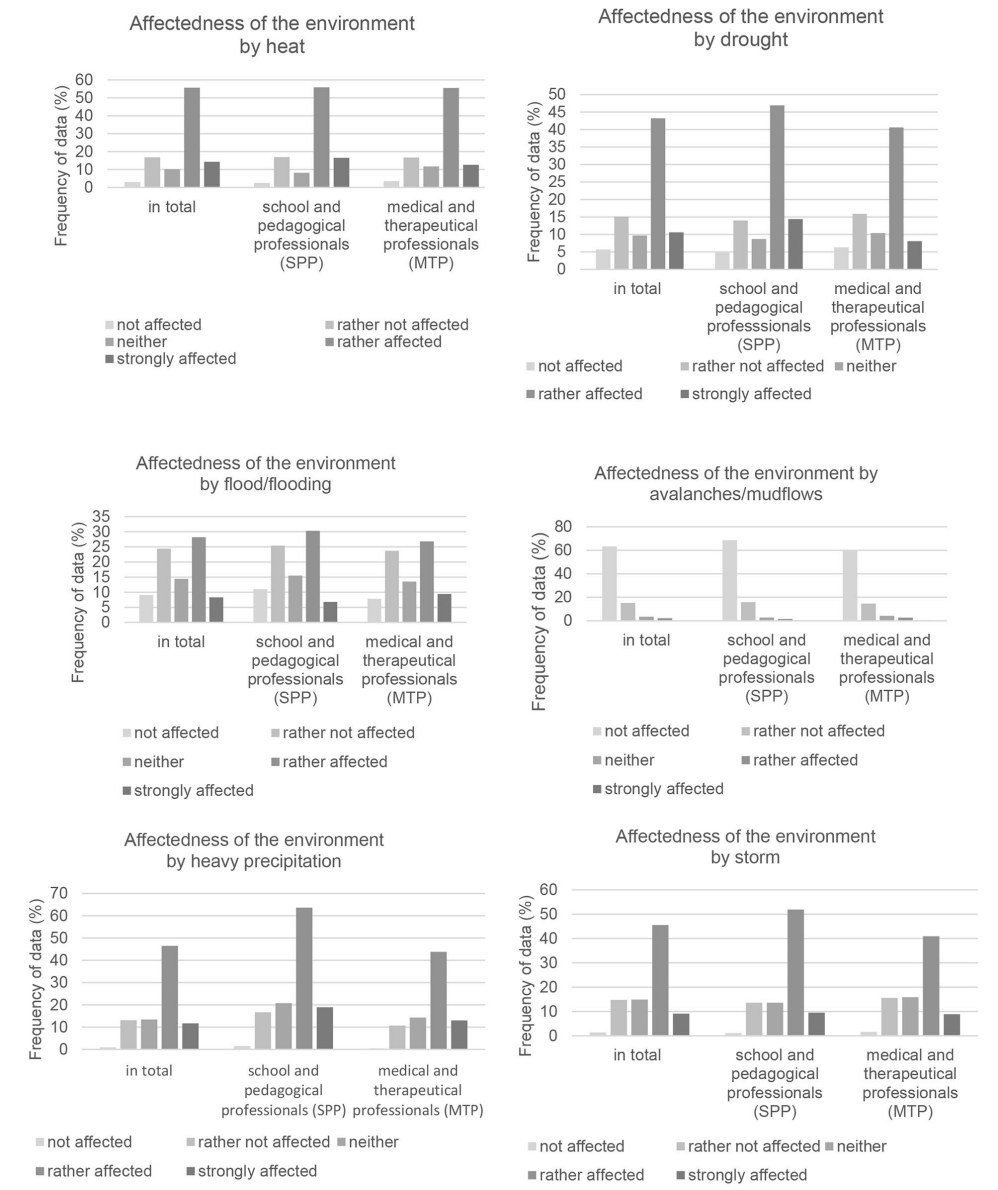
Affectedness of the personal environment by the EWE heat, drought, storm, heavy precipitation, flood/flooding, avalanches/mudflows; response behavior subdivided into the occupational groups

When asked about how affected their personal environment is by six different EWE, the majority of respondents indicated “rather affected” for heat, drought, storms, and heavy precipitation (see Fig. 2). For the EWE flood and flooding, there is a large group of respondents who checked “rather affected” and a roughly equal group who checked “rather not affected.” Only a few people were affected by avalanches and mudflows. Overall, there was not much difference in the frequency distribution between the two occupational groups.

#### Population density of the work environment

**Fig. 3:**
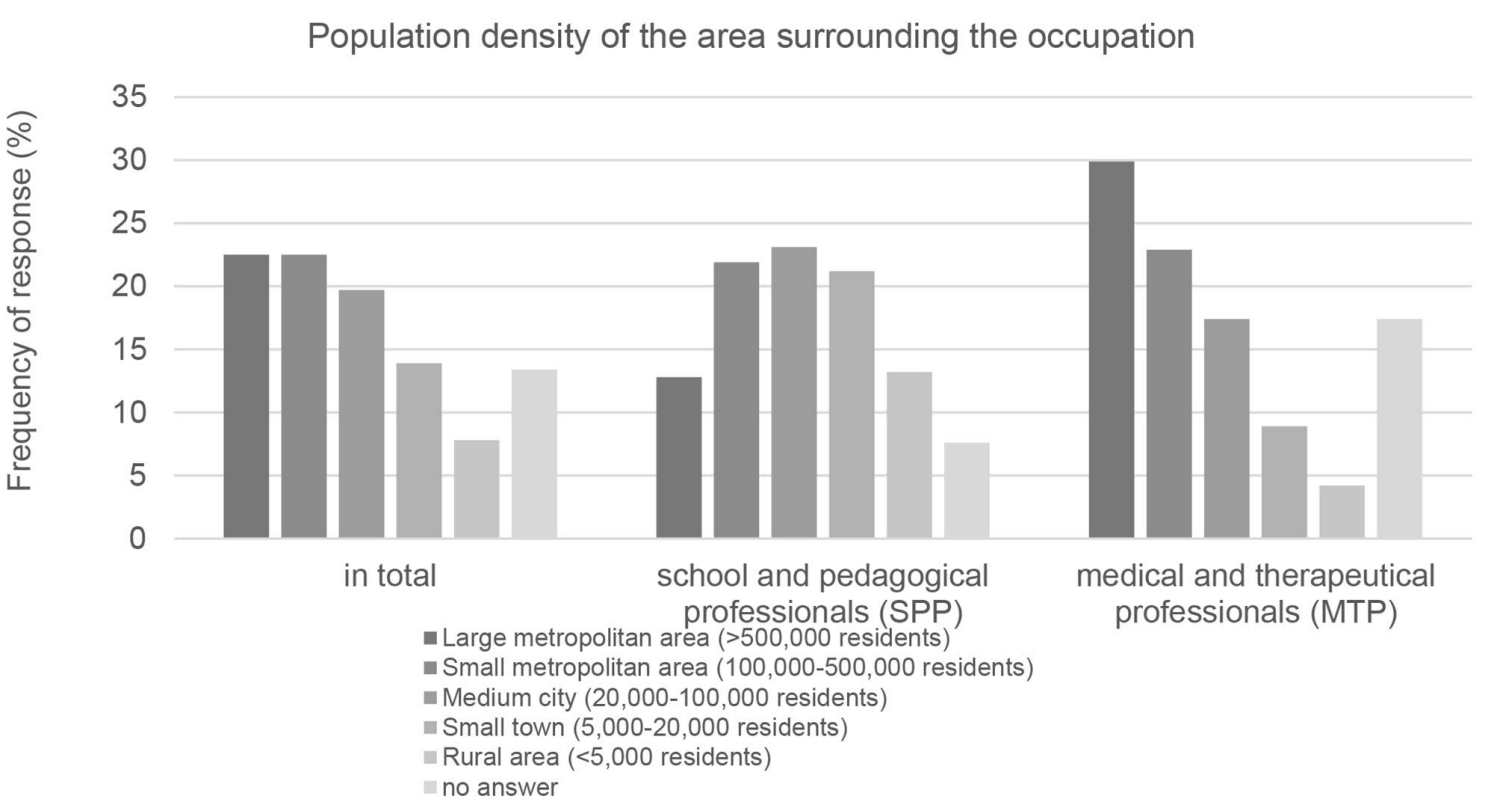
Population density of the work environment; response behavior subdivided into occupational groups

The largest group of respondents indicated that they work in a large or small metropolitan area (see Fig. 3). There were large differences between the professional groups surveyed: MTP reported working mainly in large and small metropolitan areas. SPP, on the other hand, made up the majority of the respondents with a job in rural areas and small towns.

### Results of the regression analyses

**Hypothesis a)**

*Respondents perceive the relevance of mental health impairments of children and adolescents associated with EWE when their environment is more affected by EWE.*

**Fig. 4:**
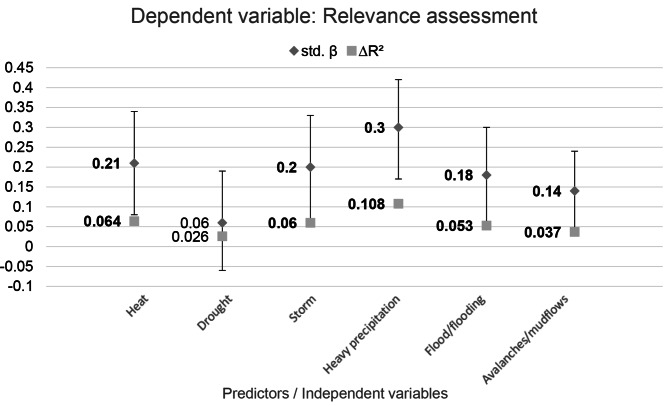
: Multiple linear regressions with 95% confidence intervals: relevance assessment as a function of being affected by the different EWE. Results printed in bold are statistically significant (*p* < 0.05)

The test conducted in the first step showed no significant effects for the control variables age and gender for any of the EWE. The results of the multiple linear regression show that being affected by EWE has a significant effect in terms of relevance assessment for all EWE except drought (see Fig. 4). Thus, for the example of being affected by heat, the relevance score increases by the value of the standardized β, by 0.21 points per standard deviation (SD). The variance increase, the R², shows a small effect for all EWE. The chi-square difference test shows that the variance growth is significant in all EWE except drought. Therefore, hypothesis a) can be accepted.

**Hypothesis b)**

*Respondents who work in an urban environment rate the relevance of the mental health impairments of children and adolescents associated with EWE as greater than those who work in a rural environment.*

**Fig. 5:**
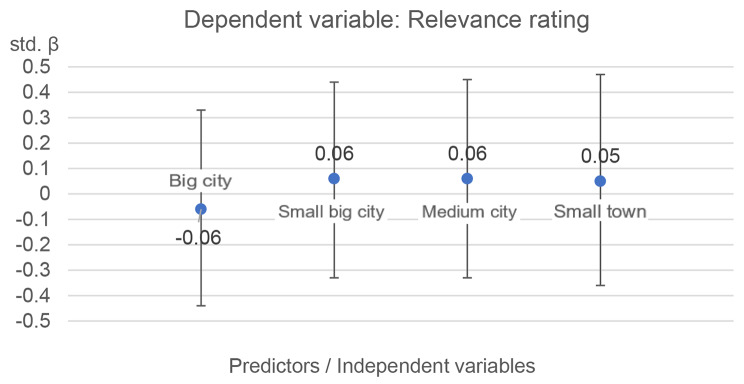
Multiple linear regressions with 95% confidence interval: Relevance assessment as a function of the population density of the work environment. Rural area is used as baseline

Again, the control variables of age and gender tested in the first step did not reveal significant effects for any of the EWE. The results of the multiple linear regressions with rural area as the baseline show that the population density of the work environment has no significant effect on the relevance assessment (see Fig. 5). Testing the results using the chi-square difference test also showed no significant results. Thus, hypothesis B has to be rejected.

The calculations of the multiple linear regressions on the hypotheses and the chi-square difference tests can be found in the additional file 1.

## Discussion

### Summary

The results of the descriptive statistics show that the majority of the participants (about 70%) rate the EWE-associated impairment of children’s and adolescents’ mental health due to EWE as relevant and only a small part (about 5%) rate it as not relevant at all. The extent to which participants state the affectedness of their personal environment by EWE varies greatly: The majority of respondents feel affected by heat (about 70%), heavy precipitation (about 58%), storms (about 54%) and droughts (about 53%). A little more than one third (about 36%) feels affected by floods / flooding and only a small part of the participants (about 3%) feel affected by avalanches / mudflows. MTP made up the majority of respondents from large and small metropolitan areas, while SPP largely reported working in rural areas and small towns.

The MLRs testing the two hypotheses found that the affectedness by EWE heat, storm, heavy precipitation, flood / flooding, and avalanches / mudflows made a small but significant contribution to explaining the variance in relevance assessments. The results for testing the influence of population density were not significant. Accordingly, no evidence could be found that an urban environment increases the relevance rating.

### Interpretation

#### Affectedness of the personal environment by EWE

The results of our study confirm the statements of the existing literature that the affectedness of EWE is an influencing factor for the relevance rating [26–29]. However, it is questionable why being affected by the EWE drought cannot significantly help to explain the relevance assessment of the mental health impairment of children and adolescents due to EWE in Germany, even though more than half of the respondents feel affected by droughts. One possible reason for this is that drought differs from the other EWEs in that it is not an acute event, but rather a gradual one, and the magnitudes are not seen from the beginning. This could mean that the environmental changes caused by the drought are not tangible and directly felt by the affected population to the same extend as the other EWE. This explanation is in line with the assumptions expressed by Gidden’s Paradox, which states that people do not become aware of the consequences of climate change until they become tangible and visible [30]. Gaining this awareness is necessary for a high relevance rating and thus the precondition for changing one’s own behavior, implementing adaptation measures and planning intervention strategies [10, 25, 26].

#### Personal environment

That the living and working environment can affect the relevance rating of climate change impacts has been described in foreign studies [31–34]. Both in our study and in a study by Kuckartz (2007), this could not be confirmed for the German population [35]. One possible explanation could be that the studies cited in the previous sentences surveyed the population density of the general personal environment and in our study only the population density of the work environment was measured. People who work in urban environments do not necessarily live there, which may be reflected in the relevance rating. Another possible reason for this discrepancy could be that the respondents all have a high level of education and therefore know how to obtain information from reliable sources regardless of their residential environment. In addition, the academics interviewed do not portray a representative sample of both the rural and urban population, which could explain why no influence could be detected.

### Limitations

According to statistics from the German Medical Association as of December 31st in 2020, there are currently 15,732 working physicians in pediatric and adolescent medicine [39]. This results in a sample size of 375 participants at a confidence level of 95% and a margin of error of 5% to achieve representative results. Comparably, 2,613 working physicians in child and adolescent psychiatry and psychotherapy and the same confidence level and margin of error result in a sample of 335 participants [39]. The calculation with the same values results in a case number of 381 for 44,158 working general practitioners [39]. After applying the same calculation to 790,605 / 799,314 working teachers in the school year 2020/2021 / 2021/2022, the same values of confidence level and margin of error result in a representative case number of 384 [40]. Despite the large response, the sample size of this study was not representative due to the many different occupational groups.

In addition, it can be assumed that people who are more concerned with the effects of climate change show a higher motivation to answer a questionnaire on the topic. Among other things, this would lead to a higher average relevance rating and distort the results. In addition, it is possible that the respondents’ high level of education and the resulting high level of climate change awareness make them rate the relevance of mental health impairments of children and adolescents as higher due to EWE [35].

Overall, the generalizability of the results must be assumed as being limited.

## Conclusion

Children and adolescents will have to bear a large part of the consequences of climate change today and in the future. The caretaking professionals have a special responsibility for children and adolescents’ health. According to their professional code of conduct, doctors are supposed to stand up for the preservation of the foundations of life and human health, and teachers are supposed to protect their entrusted students from harm.

A high relevance assessment of in our case the impact of EWE on children’s and adolescent’s mental health is an important precondition for changing one’s own behavior, implementing adaptation measures and planning intervention strategies. All this is necessary to protect children and young people from the mental health effects of EWE.

However, the described influence of being affected by EWE should not be seen as the sole cause of a higher relevance rating. This is because the affectedness of the environment by EWE is likely only one part of a multi-causal web of influencing factors that lead to a more realistic assessment of the risk of EWE on the mental health of children and adolescents.

Deeper knowledge of the factors influencing the relevance assessment is necessary to address the key points in risk communication in order to increase risk awareness. However, it is even more important to investigate the effects of the consequences of EWE on mental health of children and adolescents, especially in Germany due to the lack of data. With a sound data base of clear statistical figures on the prevalence of mental health impairments in children and adolescents in consequence of EWE, risk communication could be made clearer and more effective. Future studies should focus on these issues first.

### Electronic supplementary material

Below is the link to the electronic supplementary material.

Supplementary Material 1

Supplementary Material 2

## Data Availability

The datasets generated and analysed during the current study are not publicly available due to data protection regulations in Germany but are available from the corresponding author on reasonable request. The used code is available in the additional file 1.

## Abbreviations

EWE: extreme weather event
MLR: multiple linear regression
MTP: medical and therapeutic personnel
SPP: school and pedagogical personnel
