# Supplementary material for: A Germany-wide survey of caregiving professionals on climate change and mental health of children and adolescents - factors influencing their relevance rating of extreme weather event associated mental health impairments
Source: BMC Public Health. 2024 Jan 8;24:120. doi: 10.1186/s12889-023-17576-6 (PMC10775442; doi:10.1186/s12889-023-17576-6)
Supplement: Supplementary file 1 — Supplementary Material 1 [file 12889_2023_17576_MOESM1_ESM.docx]

# Additional file 1

## to the article:

*A Germany-wide survey of caregiving professionals on climate change and mental health of children and adolescents - Factors influencing their relevance rating of extreme weather event associated mental health impairments*

Annika Hieronimi ^1,*^, Fiona O’Reilly ^1^, Michael Schneider ^2^, Inga Wermuth ^3^, Gerd Schulte-Körne ^3^, Lena Lagally ^1^, Stephan Bose-O’Reilly ^1,4^, Erik Danay ^4^

^1^ Institute and Clinic for Occupational, Social and Environmental Medicine, University Hospital, LMU Munich, Munich, Germany

^2^ Institute of Sociology, Ludwig-Maximilians-University Munich, Munich, Germany

^3^ Department of Child and Adolescent Psychiatry, Psychosomatics and Psychotherapy, University Hospital, LMU Munich, Munich, Germany

^4^ Institute of Public Health, Medical Decision Making and Health Technology Assessment, Department of Public Health, Health Services Research and Health Technology Assessment, UMIT University of Health Sciences, Medical Informatics and Technology, Hall in Tirol, Austria

* Corresponding author

Code

### Descriptive analysis (in total and subdivided into occupational groups)

# Relevance assessment

table(dnona$Relevanz_psych_Erkrankung_EWE)

table(dnona$Relevanz_psych_Erkrankung_EWE, dnona$Berufsgruppe)

# Affectedness by EWE

table(dnona$umgebung_hitze)

table(dnona$umgebung_hitze, dnona$Berufsgruppe)

table(dnona$umgebung_Dürre)

table(dnona$umgebung_Dürre, dnona$Berufsgruppe)

table(dnona$umgebung_Sturm)

table(dnona$umgebung_Sturm, dnona$Berufsgruppe)

table(dnona$umgebung_Starkniederschläge)

table(dnona$umgebung_Starkniederschläge, dnona$Berufsgruppe)

table(dnona$umgebung_Hochwasser_Überschwemmung)

table(dnona$umgebung_Hochwasser_Überschwemmung, dnona$Berufsgruppe)

table(dnona$umgebung_lawinen_muren)

table(dnona$umgebung_lawinen_muren, dnona$Berufsgruppe)

# Population density

table(dnona$umgebung)

table(dnona$umgebung, dnona$Berufsgruppe)

# Sociodemographic data

table(dnona$alter)

table(dnona$geschlecht)

table(dnona$Berufsgruppe

### Multiple linear regressions hypothesis a)

# heat

dhypo <- dnona[c("Relevanz_psych_Erkrankung_EWE", "alter", "geschlecht", "umgebung_hitze")]

names(dhypo) <- c("Relevanz_psych_EWE", "Alter", "Geschlecht", "Hitze")

dhyp <- na.omit(dhypo)

erglm1 <- lm(Relevanz_psych_EWE ~ Alter+Geschlecht, data=dhyp)

tab_model(erglm1)

erglm2 <- lm(Relevanz_psych_EWE ~ Alter+Geschlecht+ Hitze, data=dhyp)

tab_model(erglm2)

tab_model(erglm1,erglm2,show.std = TRUE, robust = TRUE)

anova(erglm1,erglm2)

# drought

dhypo1 <- dnona[c( "Relevanz_psych_Erkrankung_EWE", "alter",

"geschlecht", "umgebung_Dürre")]

names(dhypo1) <- c( "Relevanz_psych_EWE", "Alter", "Geschlecht", "Dürre")

dhyp1 <- na.omit(dhypo1)

erglm11 <- lm(Relevanz_psych_EWE ~ Alter+Geschlecht, data=dhyp1)

tab_model(erglm11)

erglm21 <- lm(Relevanz_psych_EWE ~ Alter+Geschlecht+ Dürre , data=dhyp1)

tab_model(erglm21)

tab_model(erglm11,erglm21,show.std = TRUE, robust = TRUE)

anova(erglm11,erglm21)

# flood / flooding

dhypo2 <- dnona[c("Relevanz_psych_Erkrankung_EWE", "alter",

"geschlecht", "umgebung_Hochwasser_Überschwemmung")]

names(dhypo2) <- c("Relevanz_psych_EWE", "Alter", "Geschlecht", "Hochwasser_Überschwemmung")

dhyp2 <- na.omit(dhypo2)

erglm12 <- lm(Relevanz_psych_EWE ~ Alter+Geschlecht, data=dhyp2)

tab_model(erglm12)

erglm22 <- lm(Relevanz_psych_EWE ~ Alter+Geschlecht+ Hochwasser_Überschwemmung, data=dhyp2)

tab_model(erglm22)

tab_model(erglm12,erglm22,show.std = TRUE, robust = TRUE)

anova(erglm12,erglm22)

# avalanches/mudflows

dhypo3 <- dnona[c( "Relevanz_psych_Erkrankung_EWE", "alter",

"geschlecht", "umgebung_lawinen_muren")]

names(dhypo3) <- c( "Relevanz_psych_EWE", "Alter", "Geschlecht", "Lawinen_Muren")

dhyp3 <- na.omit(dhypo3)

erglm13 <- lm(Relevanz_psych_EWE ~ Alter+Geschlecht, data=dhyp3)

tab_model(erglm13)

erglm23 <- lm(Relevanz_psych_EWE ~ Alter+Geschlecht+ Lawinen_Muren, data=dhyp3)

tab_model(erglm23)

tab_model(erglm13,erglm23,show.std = TRUE, robust = TRUE)

anova(erglm13,erglm23)

# heavy precipitation

dhypo4 <- dnona[c("Relevanz_psych_Erkrankung_EWE", "alter",

"geschlecht", "umgebung_Starkniederschläge")]

names(dhypo4) <- c( "Relevanz_psych_EWE", "Alter", "Geschlecht", "Starkniederschläge")

dhyp4 <- na.omit(dhypo4)

erglm14 <- lm(Relevanz_psych_EWE ~ Alter+Geschlecht, data=dhyp4)

tab_model(erglm14)

erglm24 <- lm(Relevanz_psych_EWE ~ Alter+Geschlecht+ Starkniederschläge , data=dhyp4)

tab_model(erglm24)

tab_model(erglm14,erglm24 ,show.std = TRUE, robust = TRUE)

anova(erglm14,erglm24)

# storm

dhypo5 <- dnona[c("Relevanz_psych_Erkrankung_EWE", "alter",

"geschlecht", "umgebung_Sturm")]

names(dhypo5) <- c("Relevanz_psych_EWE", "Alter", "Geschlecht", "Sturm")

dhyp5 <- na.omit(dhypo5)

erglm15 <- lm(Relevanz_psych_EWE ~ Alter+Geschlecht, data=dhyp5)

tab_model(erglm15)

erglm25 <- lm(Relevanz_psych_EWE ~ Alter+Geschlecht+ Sturm, data=dhyp5)

tab_model(erglm25)

tab_model(erglm15,erglm25,show.std = TRUE, robust = TRUE)

anova(erglm15,erglm25)

### Multiple linear regressions hypothesis b)

dnona$Umgebungf <- as.factor(dnona$umgebung)

dnona$UmgebungR <- relevel(dnona$Umgebungf, ref = "Ländliche Umgebung (< 5.000 Einwohner)")

dhypo6 <- dnona[c("Relevanz_psych_Erkrankung_EWE", "alter", "geschlecht", "UmgebungR")]

names(dhypo6) <- c("Relevanz_psych_EWE", "Alter", "Geschlecht", "Populationsdichte")

table(dnona$umgebung )

dhyp6 <- na.omit(dhypo6)

erglm6 <- lm(Relevanz_psych_EWE ~ Alter + Geschlecht , data = dhyp6)

erglm61 <- lm(Relevanz_psych_EWE ~ Alter + Geschlecht + Populationsdichte, data = dhyp6)

tab_model(erglm6, erglm61)

anova(erglm6, erglm61)

Results

### Results from multiple linear regressions hypothesis a)

| Predictors | β | 95% $\mathrm{CI}_{\beta}$ | | *p* | *∆R*² | Observations |
| --- | --- | --- | --- | --- | --- | --- |
|  |  | *LL* | *UL* |  |  | N |
| Heat | 0.21 | 2.08 | 3.24 | **.002** | .064 | 284 |
| Drought | 0.06 | -0.07 | 0.20 | .331 | .026 | 280 |
| Flood and flooding | 0.18 | 0.05 | 0.29 | **.007** | .053 | 281 |
| Avalanches and mudflows | 0.14 | 0.06 | 0.35 | **.005** | .037 | 281 |
| Heavy precipitation | 0.30 | 0.22 | 0.55 | **<.001** | .108 | 283 |
| Storm | 0.20 | 0.09 | 0.39 | **.002** | .060 | 285 |

*Note:* β = standardized β; $\mathrm{CI}_{\beta}$= Confidence interval for β; *LL* = lower limit; UL = Upper limit; significant *p* < .05; *∆R*² = Increase in variance.

### Results from multiple linear regressions hypothesis b)

| Predictors / Independent variables | Std. β | 95% $\mathrm{CI}_{\beta}$ | | *p* | | observations |
| --- | --- | --- | --- | --- | --- | --- |
|  |  | LL | UL | |  | N |
| Big city | -0.06 | -0.44 | 0.33 | | .770 | 537 |
| Small big city | 0.06 | -0.33 | 0.44 | | .769 | 537 |
| Medium city | 0.05 | -0.36 | 0.47 | | .805 | 537 |
| Small town | 0.06 | -0.33 | 0.45 | | .761 | 537 |

*Note:* β = standardized β; $\mathrm{CI}_{\beta}$= Confidence interval for β; *LL* = lower limit; UL = upper limit;
significant *p* < .05.
