# Supplementary material for: A Germany-wide survey of caregiving professionals on climate change and mental health of children and adolescents - factors influencing their relevance rating of extreme weather event associated mental health impairments
Source: BMC Public Health. 2024 Jan 8;24:120. doi: 10.1186/s12889-023-17576-6 (PMC10775442; doi:10.1186/s12889-023-17576-6)
Supplement: Supplementary file 2 — Supplementary Material 2 [file 12889_2023_17576_MOESM2_ESM.docx]

# Additional file 2

## to the article:

*A Germany-wide survey of caregiving professionals on climate change and mental health of children and adolescents - Factors influencing their relevance rating of extreme weather event associated mental health impairments*

Annika Hieronimi ^1,*^, Fiona O’Reilly ^1^, Michael Schneider ^2^, Inga Wermuth ^3^, Gerd Schulte-Körne ^3^, Lena Lagally ^1^, Stephan Bose-O’Reilly ^1,4^, Erik Danay ^4^

^1^ Institute and Clinic for Occupational, Social and Environmental Medicine, University Hospital, LMU Munich, Munich, Germany

^2^ Institute of Sociology, Ludwig-Maximilians-University Munich, Munich, Germany

^3^ Department of Child and Adolescent Psychiatry, Psychosomatics and Psychotherapy, University Hospital, LMU Munich, Munich, Germany

^4^ Institute of Public Health, Medical Decision Making and Health Technology Assessment, Department of Public Health, Health Services Research and Health Technology Assessment, UMIT University of Health Sciences, Medical Informatics and Technology, Hall in Tirol, Austria

* Corresponding author

Descriptive analysis of sociodemographic data

|  | | | N | N in % |
| --- | --- | --- | --- | --- |
| Frequencies of the given age groups | | | 648 | 100 |
| 34 years or younger | | 111 | 17.1 |  |
| 35 to 39 years | | 57 | 8.8 |  |
| 40 to 49 years | | 126 | 19.4 |  |
| 50 to 59 years | | 172 | 26.5 |  |
| 60 to 65 years | | 69 | 10.6 |  |
| over 65 years | | 25 | 3.9 |  |
| Not indicated | | 88 | 13.6 |  |
| Gender | | | 648 | 100 |
| Female | 383 | 59.1 |  |  |
| Male | 170 | 26.2 |  |  |
| Divers | 2 | 0.3 |  |  |
| Not indicated | 93 | 14.4 |  |  |
| Occupational groups (filter question) | | | 648 | 100 |
| medical and therapeutic professionals | 384 | 59.3 |  |  |
| school and pedagogical professionals | 264 | 40.7 |  |  |
|  | | |  |  |

Table 1. Sociodemographic data; N = 648.

Frequencies of postal codes among respondents

| **PC** | **F** | **PC** | **F** | **PC** | **F** | **PC** | **F** |
| --- | --- | --- | --- | --- | --- | --- | --- |
| 00 | 3 | 27 | 7 | 50 | 5 | 74 | 4 |
| 02 | 1 | 28 | 4 | 51 | 2 | 75 | 3 |
| 04 | 13 | 29 | 2 | 52 | 9 | 76 | 4 |
| 05 | 2 | 30 | 5 | 53 | 4 | 77 | 2 |
| 06 | 5 | 31 | 2 | 54 | 3 | 78 | 3 |
| 07 | 25 | 32 | 2 | 55 | 16 | 79 | 6 |
| 09 | 2 | 33 | 8 | 56 | 1 | 80 | 23 |
| 10 | 15 | 35 | 3 | 58 | 1 | 81 | 22 |
| 12 | 1 | 36 | 2 | 59 | 4 | 82 | 5 |
| 13 | 3 | 37 | 5 | 60 | 4 | 83 | 3 |
| 14 | 8 | 38 | 3 | 61 | 1 | 85 | 23 |
| 15 | 1 | 39 | 3 | 63 | 12 | 86 | 4 |
| 17 | 4 | 40 | 8 | 65 | 6 | 87 | 3 |
| 18 | 1 | 41 | 2 | 66 | 25 | 88 | 2 |
| 19 | 5 | 42 | 3 | 67 | 3 | 89 | 5 |
| 20 | 5 | 44 | 5 | 68 | 12 | 90 | 5 |
| 21 | 4 | 45 | 7 | 69 | 4 | 91 | 3 |
| 22 | 4 | 46 | 10 | 70 | 6 | 92 | 2 |
| 24 | 7 | 47 | 12 | 71 | 3 | 93 | 4 |
| 25 | 2 | 48 | 5 | 72 | 4 | 94 | 5 |
| 26 | 2 | 49 | 4 | 73 | 5 | 95 | 1 |

Table 2. Frequencies of postal codes among respondents (PC: first two digits of the postal code, F: frequency)
